# Methodological considerations for the establishment of epidemiological cutoff values for *Sporothrix* species using CLSI broth microdilution

**DOI:** 10.1128/aac.00533-26

**Published:** 2026-06-24

**Authors:** Guanghui Chen, Yang Zou, Keqian Chen

**Affiliations:** 1 Department of Clinical Pharmacy, The Central Hospital of Xiangtan (The Affiliated Hospital of Hunan University) 117752 , Xiangtan, China; University Children's Hospital Münster, Münster, Germany

**Keywords:** antifungal susceptibility testing, CLSI broth microdilution, epidemiological cutoff values, *Sporothrix*

## LETTER

We read with great interest the recent article by dos Santos et al. establishing the first CLSI-endorsed epidemiological cutoff values (ECVs) for *Sporothrix* species (1). This collaborative effort, aggregating 3,504 MIC values from 19 international laboratories, provides a critical foundation for antifungal resistance surveillance in the expanding zoonotic sporotrichosis epidemic. However, several methodological nuances warrant emphasis to guide clinical application of these ECVs.

While the authors acknowledge that revising the CLSI protocol to 30°C incubation could ensure a pure mycelial phase, we contend this limitation has more profound implications than the text suggests. Testing at 35°C induces asynchronous yeast-phase transition in *Sporothrix* spp., which the authors link to rejection of 17%–30% of MIC data sets for *Sporothrix brasiliensis* against amphotericin B and azoles (1). Because the yeast phase exhibits MICs up to eightfold lower than the mycelial phase (2, 3), morphological mixing likely skews distributions toward higher values. Consequently, the current ECVs—most notably itraconazole at 4 µg/mL for *S. brasiliensis*—may represent methodological artifacts that overestimate true wild-type upper limits. Should the community adopt 30°C testing to improve reproducibility, these ECVs will require urgent re-evaluation.

A second concern involves the tentative terbinafine ECV of 2 µg/mL proposed for *Sporothrix globosa* based on only 38 isolates. Table 2 reveals a multimodal MIC distribution with clusters at 0.5 and 1 µg/mL. Setting the ECV at 2 µg/mL would classify isolates with MICs of 1 µg/mL as wild type, despite 1 µg/mL being exceptionally high for terbinafine in filamentous fungi (the *S. brasiliensis* ECV is 0.12 µg/mL). This pattern strongly suggests cryptic resistance mechanisms segregating within the tested population. We recommend that clinicians view terbinafine MICs ≥0.5–1 µg/mL for *S. globosa* with suspicion pending testing of a larger, geographically diverse collection.

Finally, the high interlaboratory data rejection rate underscores the absence of *Sporothrix*-specific quality control strains in CLSI M38M51S. Reference ranges for *Aspergillus fumigatus* do not adequately verify proficiency in testing dimorphic fungi under contested temperature conditions. We echo the authors’ call for characterizing at least three reference strains—wild-type S. brasiliensis, wild-type *Sporothrix schenckii*, and a defined non-wild-type *S. brasiliensis* isolate—with QC ranges established at both 30°C and 35°C.

In summary, the study by dos Santos et al. represents a landmark contribution. Our comments aim to strengthen this foundation by emphasizing that current ECVs must be applied with careful attention to unresolved methodological variables. Clarifying the impact of incubation temperature and revisiting the outlier status of *S. globosa* terbinafine MICs will ensure these ECVs serve as robust tools for detecting emerging resistance in this increasingly important zoonotic pathogen.

## References

[1] dos Santos AR, Bonifaz A, Bombassaro A, Machado AC de S, Rodrigues AM, Borman AM, Chakrabarti A, Chowdary A, Rediguieri B, Johnson EM, et al.. 2026. Establishment of epidemiological cutoff values for clinically relevant Sporothrix species using CLSI-broth microdilution. Antimicrob Agents Chemother 70:e0190725. doi:10.1128/aac.01907-25 41940808 PMC13148023

[2] Trilles L, Fernández-Torres B, Dos Santos Lazéra M, Wanke B, de Oliveira Schubach A, de Almeida Paes R, Inza I, Guarro J. 2005. In vitro antifungal susceptibilities of Sporothrix schenckii in two growth phases. Antimicrob Agents Chemother 49:3952–3954. doi:10.1128/AAC.49.9.3952-3954.2005 16127080 PMC1195444

[3] do Prado CM, Spruijtenburg B, Razzolini E, Chiyo L, Santi C, Martins CA, Santacruz G, Segovia N, Brunelli JP, Cognialli RCR, et al.. 2025. Sporothrix brasiliensis treatment failure without initial elevated itraconazole MICs in felids at border of Brazil. Emerg Infect Dis 31:1783–1792. doi:10.3201/eid3109.250156 40867021 PMC12407209

